# Pyoluteorin Produced by the Biocontrol Agent *Pseudomonas protegens* Is Involved in the Inhibition of *Heterobasidion* Species Present in Europe

**DOI:** 10.3390/pathogens11040391

**Published:** 2022-03-23

**Authors:** Martina Pellicciaro, Elio Padoan, Guglielmo Lione, Luisella Celi, Paolo Gonthier

**Affiliations:** Department of Agricultural, Forest and Food Sciences (DISAFA), University of Turin, 10095 Grugliasco, Italy; martina.pellicciaro@unito.it (M.P.); elio.padoan@unito.it (E.P.); luisella.celi@unito.it (L.C.); paolo.gonthier@unito.it (P.G.)

**Keywords:** biological control, antibiosis, secondary metabolites, co-culture, 2,4-diacetylphloroglucinol, pyrrolnitrin, HPLC-MS, Proradix^®^, cell-free filtrate, forest pathogen

## Abstract

*Pseudomonas protegens* (strain DSMZ 13134) is a biocontrol agent with promising antagonistic activity hinging on antibiosis against the fungal forest pathogens *Heterobasidion* spp. Here, by using High-Performance Liquid Chromatography coupled to Mass Spectrometry (HPLC-MS), we assessed whether monocultures of *P. protegens* (strain DSMZ 13134) produce the three major determinants of biocontrol activity known for the genus *Pseudomonas*: 2,4-diacetylphloroglucinol (2,4-DAPG), pyoluteorin (PLT), and pyrrolnitrin (PRN). At the tested culture conditions, we observed the production of PLT at concentrations ranging from 0.01 to 10.21 mg/L and 2,4-DAPG at a concentration not exceeding 0.5 mg/L. Variations of culture conditions involving culture medium, incubation temperature, and incubation period had no consistent influence on PLT production by the bacterium. Assays using culture medium amended with PLT at the same concentration of that present in cell-free filtrate of the bacterium, i.e., 3.77 mg/L, previously documented as effective against *Heterobasidion* spp., showed a remarkable activity of PLT against genotypes of all the four *Heterobasidion* species present in Europe, including the non-native invasive *H. irregulare*. However, such antifungal activity decreased over time, and this may be a constraint for using this molecule as a pesticide against *Heterobasidion* spp. When the bacterium was co-cultured in liquid medium with genotypes of the different *Heterobasidion* species, an increased production of PLT was observed at 4 °C, suggesting the bacterium may perform better as a PLT producer in field applications under similar environmental conditions, i.e., at low temperatures. Our results demonstrated the role of PLT in the inhibition of *Heterobasidion* spp., although all lines of evidence suggest that antibiosis does not rely on a single constitutively produced metabolite, but rather on a plethora of secondary metabolites. Findings presented in this study will help to optimize treatments based on *Pseudomonas protegens* (strain DSMZ 13134) against *Heterobasidion* spp.

## 1. Introduction

A variety of Plant Growth-Promoting Rhizobacteria (PGPR) exhibit antagonistic effects against plant pathogens via nutrient competition, induced resistance, priming, and antibiosis [1,2,3]. *Pseudomonas* spp. have been widely studied for their biocontrol potential [1,2,4]. They show antagonistic effects against a wide range of fungal plant pathogens, including ascomycetes, basidiomycetes, and mitosporic fungi [1,2,3]. The genus *Pseudomonas* is reported to inhibit pathogens by antibiosis through the secretion of secondary metabolites [1,2,4,5,6]. Different *Pseudomonas* species secrete a plethora of secondary metabolites with antibiotic activity, including pyoluteorin (PLT), pyrrolnitrin (PRN), 2,4-diacetylphloroglucinol (2,4-DAPG), phenazines, cyclic lipopeptides, and volatile compounds [1,2,3,6]. Based on genomic information, other undetected secondary metabolites could also be secreted [4].

Diseases caused by the fungal pathogens *Heterobasidion* spp. include destructive root and butt rots in coniferous forests of the Northern Hemisphere [7]. Members of the species complex *H. annosum* (Fr.) Bref. *sensu lato* (*s.l*.) are native to Europe., including *H. abietinum* Niemelä & Korhonen, *H. annosum sensu stricto* (*s.s*.) (Fr.) Bref., hereafter referred to as *H. annosum*, and *H. parviporum* Niemelä & Korhonen which are mainly associated with *Abies alba* Mill., *Pinus* spp., and *Picea abies* (L.) Karst., respectively [7]. In central Italy, stone pine (*P. pinea* L.) stands along the west coastline are threatened by the invasive North American *H. irregulare* Garbel. & Otrosina [8]. Following a pest risk analysis [9], this non-native *Heterobasidion* species is currently recommended for regulation under the European and Mediterranean Plant Protection Organization (EPPO) A2 list. Disease management implies treating freshly cut stumps with either chemical or biological products to prevent stump infection by spores and subsequent spreading of the fungi to neighboring healthy trees through root contacts [7].

Our study focuses on the PGPR *Pseudomonas protegens* (strain DSMZ 13134), which is the active component of the bio fungicide Proradix^®^ (SP Sourcon Padena GmbH, Tübingen, Germany) currently commercialized against black scurf caused by *Rhizoctonia solani* J.G. Kühn and silver scab caused by *Helmintosporium solani* Durieu & Mont. on potatoes and other tubers. Previous laboratory and field studies had shown the ability of this PGPR to display significant antagonistic effects towards the fungal forest pathogens *Heterobasidion* spp., making it a candidate biocontrol agent against the *Heterobasidion* species [10,11,12,13]. Moreover, the cell-free filtrate (CFF) of the same bacterium displayed the ability to inhibit both mycelial growth (100% inhibition) and conidial germination (99% inhibition) of the pathogen in vitro [12] and performed even better than the bio fungicide Proradix^®^ in experiments conducted in controlled conditions and in the forest on stumps of several host tree species [12,13]. It has been shown that antibiosis is likely the main mechanism of action of *P. protegens* (strain DSMZ 13134) against *Heterobasidion* spp. [12,13], as well as towards other fungal pathogens [14]. The secondary metabolites with antifungal activity present in the CFF are the putative determinants of both Proradix^®^ and CFF efficacy, but those compounds and their mechanisms of action remain completely unknown. Knowing the mechanisms of action of a biocontrol agent can help with the optimization of the disease control, but it is also required for registration where risks for humans and the environment, including risks for resistance development, have also to be indicated [4,15]. Regulation in the EU makes a distinction between biocontrol agents able to produce secondary metabolites with antimicrobial activity in situ and such compounds in product without living cells of the biocontrol agent [4]. 

This study investigated the formation and the concentration of three of the major known determinants of biocontrol activity of *Pseudomonas* spp., namely 2,4-DAPG, PLT and PRN [1,2,3,6,16] in the CFF of *P. protegens* (strain DSMZ 13134) used as both an amendment in medium inhibiting *Heterobasidion* spp. in vitro and a stump treatment in a previous field study [12,13]. The antifungal activity of the selected secondary metabolites present in the CFF, and its contribution to the antifungal activity of the raw CFF, were evaluated in vitro at different times against the four *Heterobasidion* spp. currently occurring in Europe, including the non-native invasive *H. irregulare*. In addition, we assessed whether the variety and yield of such metabolites could be enhanced by acting on culture conditions (culture medium, incubation temperature, and incubation period). Our ultimate aim was to explore whether the production of these compounds by *P. protegens* (strain DSMZ 13134) is temperature-controlled during the interaction with *Heterobasidion* species. This could help to predict the production of metabolites when the bacterium (i.e., Proradix^®^) is applied on stump surfaces in infested sites under different environmental conditions.

## 2. Results

### 2.1. Presence and Concentration of Selected Secondary Metabolites in the CFF of P. protegens (Strain DSMZ 13134)

To determine whether the selected secondary metabolites are produced by *P. protegens* (strain DSMZ 13134), we generated a monoculture of the bacterium in Luria–Bertani (LB) broth for 24 h at 25 °C. The CFF of the monoculture was obtained by filtering the bacterial culture aseptically through a 0.22 μm filter membrane. Chemical analysis of CFF through High-Performance Liquid Chromatography coupled to Mass Spectrometry (HPLC-MS) allowed us to determine the presence of PLT at the concentration of 3.77 mg/L (Table 1). Neither 2,4-DAPG nor PRN were detected in the CFF of *P. protegens* (strain DSMZ 13134) (Table 1).

### 2.2. Effects of PLT on Mycelial Growth of Heterobasidion spp.

**Figure 1:**
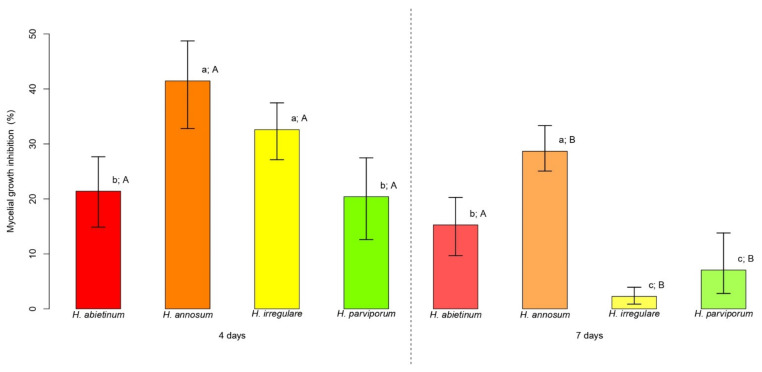
Mycelial growth inhibition (MGI) comparisons. For each time point (4 and 7 days) bars indicate the average MGI displayed by the four species of *Heterobasidion*. Error bars refer to the lower and upper bounds of the 95% confidence interval. Above each bar, lowercase letters refer to the comparisons of average MGI values among *Heterobasidion* species for each time point, while upper case letters refer to the comparison of the two time points for the same species. Different letters indicate significant differences (*p <* 0.05).

PLT at the concentration of 3.77 mg/L (consistent with that present in CFF) was assayed for mycelial growth inhibition (MGI in %) of *Heterobasidion* genotypes. The average MGI of *Heterobasidion* spp. ranged from 20.4% (12.6–27.5% 95% CI) to 41.5% (32.8–48.7% 95% CI) at 4 days, and from 2.3% (0.9–3.9% 95% CI) to 28.7% (25.1–33.3% 95% CI) at 7 days (Figure 1). At 4 days, *H. annosum* and *H. irregulare* attained average values of MGI over 40% and 30%, respectively, which were significantly higher than those observed for *H. abietinum* and *H. parviporum*, both at around 20% (*p <* 0.05) (Figure 1). *H. annosum* was the most inhibited species at 7 days with an average MGI around 30%, a value twofold higher than that attained by *H. abietinum* (*p <* 0.05) (Figure 1). At the same time point, *H. irregulare* and *H. parviporum* were significantly less inhibited than the other *Heterobasidion* species (*p <* 0.05), attaining average values of MGI around 2 and 7%, respectively (Figure 1). From the first to the second time point, a reduction in the average MGI was observed for all *Heterobasidion* species achieving approximately −6% for *H. abietinum* (*p >* 0.05), −13% for *H. annosum* and *H. parviporum* (*p <* 0.05), and −30% for *H. irregulare* (*p <* 0.05) (Figure 1).

### 2.3. Effects of Culture Conditions on Secondary Metabolites Production by Monocultures of P. protegens (Strain DSMZ 13134) 

To evaluate how cultural parameters may affect secondary metabolites production in *P. protegens* (strain DSMZ 13134)*,* we tested a panel of monocultures of the bacterium growing at different conditions. *Pseudomonas protegens* (strain DSMZ 13134) produced PLT at concentrations ranging from 0.01 to 10.21 mg/L depending on cultural conditions (Table 1). Quantification of 2,4-DAPG was possible only in monocultures grown in LB for 24 h at 4 and 30 °C, whereas in the other culture conditions, 2,4-DAPG was under the limit of quantification (LOQ, 0.5 mg/L) or it was not detected (Table 1). Concentrations of PRN were always under the detection limit (LOD) (Table 1). 

**Figure 2:**
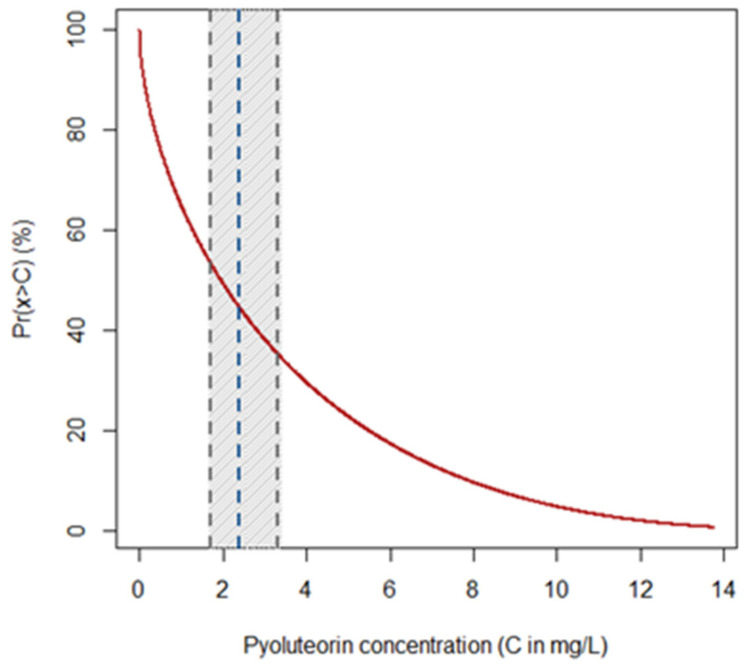
Probability of retrieving pyoluteorin (PLT) from monocultures of *P. protegens* (strain DSMZ 13134) with concentrations over a given threshold. The graph shows the thresholds C of PLT concentration (in mg/L) on the x-axis, while the y-axis reports the probability ($\mathrm{Pr}\left(x>C\right)$ in %) that *P. protegens* (strain DSMZ 13134) may release higher concentrations of the compound. The red curve quantifies the probability, while the blue dashed lines represent the average expected concentration of PLT along with the lower and upper bounds (gray dashed lines) of its 95% confidence interval (gray strip).

Statistical analysis carried out with unbiased recursive partitioning tree model showed that the concentration of PLT produced by monocultures of *P. protegens* (strain DSMZ 13134) was not significantly associated with either culture medium, incubation temperature, or incubation period (*p >* 0.05) (Table 2). The average concentration of PLT was 2.39 mg/L (1.68–3.32 mg/L 95% CI) (Figure 2).

Among the distribution types included in the Pearson system of generalized frequency curves, type I attained the lowest AIC (68.0), while all others displayed AIC values over 200. The equation of the corresponding upper tail probability distribution function obtained was:

(1) $$\mathrm{Pr}\left(x>C\right)=\overset{+\infty }{\underset{C}{\int }}\frac{1}{\left|s\right|}\frac{\Gamma \left(a+b\right)}{\Gamma \left(a\right)\Gamma \left(b\right)}{\left(\frac{x-\lambda }{s}\right)}^{a-1}{\left(1-\frac{x-\lambda }{s}\right)}^{b-1}\mathrm{dx}$$

where the probability $\mathrm{Pr}\left(x>C\right)$ that monocultures of *P. protegens* (strain DSMZ 13134) release PLT with a concentration x higher than a given threshold C is provided by the integral of the Pearson curve of type I (Figure 2). In the above equation, Γ is the Euler Gamma function, while the curve parameters obtained through maximum likelihood estimation were $a=0.589,b=2.881,\;\lambda =-3.990\cdot 10^{-13}$, and s=18.158. The curve shows that PLT is produced by monocultures of *P. protegens* (strain DSMZ 13134) (i.e., the concentration of the metabolite is higher than 0) with a probability approaching 100% (Figure 2). The same curve indicates that the probability of retrieving PLT with a concentration of up to 3.77 mg/L (i.e., mean concentration measured in the CFF) is approximately 70% (Figure 2).

### 2.4. Effects of Co-Cultures of P. protegens (Strain DSMZ 13134) with Heterobasidion spp. on Secondary Metabolites Production by the Bacterium

**Figure 3:**
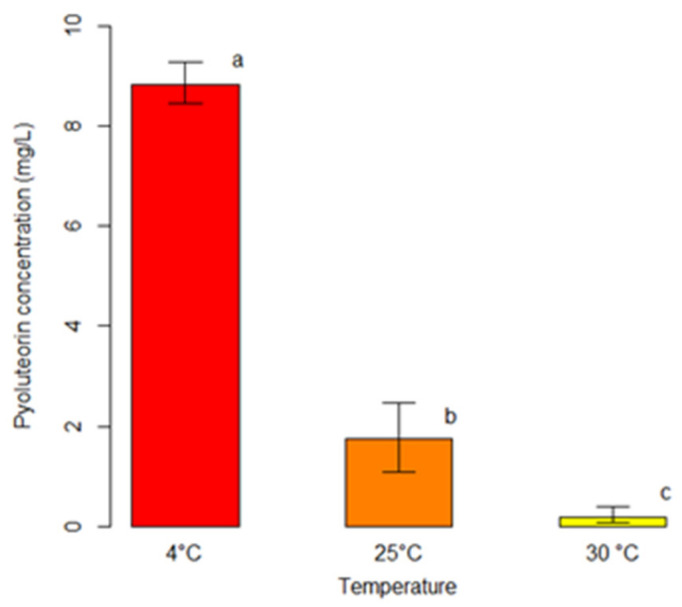
Comparison among average concentrations of pyoluteorin (PLT) produced by *P. protegens* (strain DSMZ 13134) in co-culture with *Heterobasidion* spp. at different temperatures. For each temperature, bars indicate the average concentration of PLT (mg/L). Error bars refer to the lower and upper bounds of the 95% confidence interval. Different letters above the bars indicate significant differences (*p <* 0.05).

We explored whether the production of secondary metabolites by *P. protegens* (strain DSMZ 13134) was enhanced or reduced during the interaction with *Heterobasidion* species at different temperatures. The ability to produce PLT was confirmed in co-culture experiments (Table 3; Figure 3). *Pseudomonas protegens* (strain DSMZ 13134) produced PLT at concentrations ranging from 0.07 to 9.90 mg/L in extracts of all co-cultures except one with *H. abietinum* at 30 °C (Table 3). At all tested temperatures, *P. protegens* (strain DSMZ 13134) in co-culture produced 2,4-DAPG under the LOQ (Table 3). Again, despite some variations in culture conditions, *P. protegens* (strain DSMZ 13134) failed to produce a detectable concentration of PRN (Table 3). 

The outcomes of the unbiased recursive partitioning tree model pointed out that only the incubation temperature exerted a significant effect on the production of PLT from *P. protegens* (strain DSMZ 13134) in co-culture with *Heterobasidion* spp. (*p <* 0.05) (Table 2). Conversely, the co-culture type did not display any significant association with the response variable (*p >* 0.05) (Table 2). Decreasing incubation temperatures were associated with raising concentrations of PLT ranging from an average of 0.18 mg/L at 30 °C, to 1.74 mg/L at 25 °C, up to 8.84 mg/L at 4 °C (*p <* 0.05) (Figure 3).

## 3. Discussion

Plant Growth-Promoting Rhizobacteria are ubiquitous in nature, and they may have a range of agricultural, environmental, and industrial applications. The genus *Pseudomonas* is highly appreciated for its plant growth promoting traits and because of its ability to produce a range of secondary metabolites with antibiotic activity [2,3,5,6]. Among the investigated secondary metabolites, only PLT was present in the CFF of *P. protegens* (strain DSMZ 13134). Pyoluteorin is a polyketide metabolite produced by fluorescent *Pseudomonas* known for its fungicidal, bactericidal, and herbicidal activities [2,17]. The concentration of PLT in the CFF was measured as high as 3.77 mg/L. This concentration is consistent with those found in the well-studied rhizosphere bacterium *P. protegens* Pf-5 [18,19].

PLT at the same concentration present in the CFF, i.e., 3.77 mg/L, was assayed against the mycelial growth of *Heterobasidion* spp. to determine the individual contribution of PLT to the overall efficacy of the raw CFF, which was previously shown to be 100% [12]. The probability distribution function obtained from the Pearson system showed that the concentration of PLT present in the CFF is slightly over the average, but there is a large probability (70%) that *P. protegens* (strain DSMZ 13134) can release up to this concentration. Conversely, testing higher concentrations might be not sound because only in 30% of cases the bacterium is expected to produce PLT over the threshold of 3.77 mg/L. In this study, PLT proved to have antifungal activity against all the tested *Heterobasidion* species. Yet, its efficacy decreased significantly after 7 days of incubation against all *Heterobasidion* species except *H. abietinum*. The decline in the effectiveness may be due to PLT stability, as suggested by a previous experimental study on the degradation of PLT showing that temperature, solution pH and UV irradiation had a strong influence on its degradation [20]. Interestingly, PLT was found to be unstable in acid and in alkaline solutions, and under UV irradiation, making its use as a pesticide difficult without any modification to improve the stability [20]. In vitro culture conditions might have increased PLT instability, thus reducing its efficacy against the fungal pathogens over time. Significant reductions of the efficacy of PLT in a few days may be a constraint for using this molecule as a pesticide against *Heterobasidion* spp. Because stumps remain susceptible to infection by these pathogens for a few weeks [7].

In a previous work, the same *Heterobasidion* genotypes used in this study were completely inhibited when grown on media containing the CFF, without differences in sensitivity among different species and over time [12]. Our results clearly document that PLT is not the sole compound responsible for *Heterobasidion* inhibition, as PLT alone can explain at best 41.5% of the total inhibition caused by the CFF. Consequently, all lines of evidence suggest that antibiosis does not rely on a single constitutively produced metabolite, but rather on a plethora of secondary metabolites.

The optimization of culture conditions to enhance secondary metabolites production has gained attention for its potential to implement the success of biocontrol-based products and to maximize disease control [3,4,15]. Alterations of culture conditions may have a pronounced influence on yield enhancement and de novo induction of secondary metabolites [21,22]. In the present study, *P. protegens* (strain DSMZ 13134) was efficient in producing PLT, but this production was not affected by any of the tested cultural parameters (culture medium, incubation temperature and incubation period). Although it has been reported that *P. protegens* strains can be considered as a new bacterial group able to produce 2,4-DAPG, PLT and PRN [23], it appears that our *P. protegens* strain is not capable of producing high levels of 2,4-DAPG or PRN.

Since these metabolites are usually produced and released by microorganisms in small quantities [4,16,24], although their production is strongly dependent on nutrient availability [4,15,16,25], even substantial variation of culture conditions may not be able to increase their production. The modified King B (KBM) broth, containing a larger amount of glycerol, should have indeed enhanced PLT production over 2,4-DAPG production [26,27,28]. However, this was not observed in our experiment: in fact, the substrate did not affect PLT production in our bacterial strain. Pyoluteorin and 2,4-DAPG production is interrelated in *P. protegens* and other *Pseudomonas* species, despite the independent biochemical and genetic determinants for their biosynthesis [29,30,31]. Brodhagen et al. [29] have demonstrated that PLT production is induced by positive autoregulation in *P. fluorescens* Pf-5. In addition to its autoregulatory role, PLT repressed 2,4-DAPG production [29]. Such complex interactions may have occurred in our experiments as well.

The probability distribution curve obtained from the Pearson system can be regarded as the PLT production fingerprint of *P. protegens* (strain DSMZ 13134) growing in monocultures. The curve is more informative than the simple average of the PLT concentration since it allows one to quantify the probability of retrieving up to, or more than, a given amount of the compound. More importantly, the curve clearly shows that the probability of obtaining PLT from monocultures of *P. protegens* (strain DSMZ 13134) is almost 100%, despite the fact that the concentrations can be variable and with a high probability density centered on low values. This may have a practical significance, for instance, to compare production curves of PLT or other secondary metabolites under different conditions. In fact, from an applied perspective, optimization for the production of the preferred secondary metabolites during the fermentation process of the target biocontrol agent appears as a valuable strategy toward the enhancement of the biocontrol agent itself [4,15]. This strategy is focusing on the development of a formulation that contains secondary metabolites together with living cells of the producing biocontrol agent so that the performance in field is expected to be the result of the combined effects of secondary metabolites and the potential production of additional secondary metabolites in situ [4]. This appears as a valuable alternative to the commercialization of secondary metabolites without the biocontrol agent. In fact, strictly speaking, in the absence of living cells such secondary metabolites have to be considered chemicals in the EU [4], implying an extensive characterization for risk assessment, thus increasing substantially the costs for registration [4].

In the attempt to improve our understanding of the strategies adopted by *P. protegens* (strain DSMZ 13134) in response to *Heterobasidion* spp., we assessed the effects of the interaction between the bacterium and the four *Heterobasidion* species currently occurring in Europe on the production of 2,4-DAPG, PLT and PRN at different temperatures (4 °C, 25 °C and 30 °C). Such temperatures were chosen to simulate the widest range of environmental conditions at which stump treatments are performed and hence antagonist–pathogen interaction may occur. In fact, co-culture experiments simulate natural scenarios where bacteria and fungi co-inhabit and interact in a same confined environment, in this model system a stump surface, thereby exerting intense microbial competition and interspecies crosstalk [32,33,34]. The outcomes of our co-culture experiment confirmed that *P. protegens* (strain DSMZ 13134) is capable of producing PLT. The concentration of 2,4-DAPG was again found under 0.5 mg/L, whereas no PRN production was observed. The incubation temperature of 4 °C is conducive to the production of PLT in co-culture, indicating that the antagonist–pathogen interaction at low temperatures might lead to an increased production of this antifungal compound. Among abiotic factors, temperature has been reported to affect antibiotic production by bacterial biocontrol agents [35,36,37,38,39]. However, there is not a general defined temperature at which the production of secondary metabolites by *Pseudomonas* strains is optimized because different strains have their own requirements [35,36,37,39]. Since we did not observe a similar trend in monocultures of *P. protegens* (strain DSMZ 13134), the increased production of PLT at low temperatures might be due to the interaction between the bacterium and the pathogens. The microbial co-culture is used as an experimental tool to increase the yield and variety of secondary metabolites; thus, microbial competition is deliberately provoked to activate silent metabolic pathways and/or to up-regulate gene expression [32,33,34]. Hence, we can only speculate that Proradix^®^ may perform better under similar environmental conditions, i.e., at low temperatures, thanks to its higher production of the antifungal compound PLT. An increased production of PLT on the stump surface may also be expected if the product is kept at low temperatures before use. It would be interesting to verify if a remarkably increased production of PLT, or other antifungal compounds, occurs when the bacterium grows in co-culture with saprobic, non-pathogenic fungi. It should be noted that among the possible approaches to improve biocontrol, two different scenarios based on assembled consortia of microorganisms are predicted [4]. On one side, the selection of helper strains applied to support the biocontrol agent in its establishment, survival and antagonistic activity, e.g., the production of antibiotic compounds [4]. On the other side, the application of biocontrol products consisting of different biocontrol strains combining different modes of action [4].

## 4. Materials and Methods

### 4.1. Microorganisms and Culture Conditions

*Pseudomonas protegens* (strain DSMZ 13134) was provided by SP Sourcon Padena GmbH (Tübeningen, Germany) and stored in Luria–Bertani (LB) broth containing 30% glycerol at −80 °C. Fresh cultures were started from frozen stocks for each experiment by inoculating 100 µL into LB broth (10 g of tryptone, 5 g of yeast extract, 10 g of NaCl, 1 L of H_2_O [pH 7.2]) and incubating at 25 °C for 24 h while shaking.

Fungal genotypes of each species of *H. annosum s.l.* occurring in Europe, i.e., *H. abietinum*, *H. annosum*, *H. irregulare*, and *H. parviporum*, were selected among those tested against *P. protegens* (strain DSMZ 13134) in a previous study [12] (Table 4). All genotypes of *H. annosum s.l.* were preserved in the culture collection of the University of Turin and maintained at 4 °C in pure culture on malt extract agar (MEA) (30 g of malt extract, 3 g of enzymatic digest of soybean meal, 15 g of agar-agar, 1 L of H_2_O [pH 5.6]). Inoculum for the experiments was grown by transferring a MEA plug from these maintenance cultures to fresh MEA and incubating plates at 25 °C for 7 days.

Other culture media used in this study were King B (KB) broth (20 g of proteose peptone, 1.5 g of K_2_HPO_4_, 1.5 g of MgSO_4_ ∙7H_2_O, 10 mL of glycerol, 1 L of H_2_O [pH 7.6]) [40] and a KBM broth (20 g of proteose peptone, 2.5 g of K_2_HPO_4_, 6 g of MgSO_4_· 7H_2_O, 15 mL of glycerol, 1 L of H_2_O [pH 7.6]).

### 4.2. Production of Monocultures of P. protegens (Strain DSMZ 13134) for the Quantification of 2,4-DAPG, PLT and PRN

A set of monocultures of *P. protegens* (strain DSMZ 13134) was subjected to different culture conditions to determine whether cultural parameters may affect secondary metabolite production. Monocultures were grown in the dark with constant shaking (100 rpm) in conical flasks (250 mL) containing 100 mL of LB, KB, or KBM broth. Previous studies had shown that LB broth was conducive to the production of an array of secondary metabolites able to inhibit *Heterobasidion* mycelial growth and conidial germination [12], leading to a reduction in both the colonized area and incidence of *Heterobasidion* on stumps [13]. King B broth-based media are routinely used in laboratory to enhance production of secondary metabolites in fluorescent *Pseudomonas* strains [6,29,41,42]. 

An aliquot (1 mL) of the fresh culture of *P. protegens* (strain DSMZ 13134) was inoculated in each conical flask. Monocultures were incubated at 4, 25, and 30 °C and harvested after 24 h and 7 days of incubation. Incubation temperatures of 4 and 30 °C were chosen to test the influence of low and high temperature stresses on secondary metabolites production by *P. protegens* (strain DSMZ 13134). An incubation temperature of 25 °C and harvesting at 24 h of growth were the conditions used in the previous studies, where the CFF was obtained by culturing *P. protegens* (strain DSMZ 13134) in LB broth with constant shaking at 25 °C for 24 h (OD_600_ of 1.1) [12,13]. The 7-day harvesting period was chosen because secondary metabolism usually occurs at the late growth phase of the producing microorganisms [43]. For each culture medium (LB, KB, and KBM broth) and temperature (4, 25, and 30 °C), triplicate samples were established. 

Monocultures were filtered to obtain a sample free from bacterial cells for the analysis by HPLC-MS. Cells were pelleted by centrifugation at 4000 rpm for 10 min, and the supernatant was filtered aseptically through a 0.22 μm filter membrane.

### 4.3. Production of Co-Cultures of P. protegens (Strain DSMZ 13134) and Heterobasidion spp. for the Quantification of 2,4-DAPG, PLT and PRN

A set of co-cultures of *P. protegens* (strain DSMZ 13134) and *Heterobasidion* spp. was subjected to different temperatures to explore how the production of 2,4-DAPG, PLT and PRN by *P. protegens* (strain DSMZ 13134) was modulated during the interaction between the bacterium and the fungal pathogens. To prepare co-cultures of *P. protegens* (strain DSMZ 13134) and *Heterobasidion* spp., 250 mL conical flasks were used, containing 100 mL of KB broth. 

For each species of *H. annosum s.l.*, the genotype displaying the highest growth rates in antagonism assays conducted previously [12] was selected for this experiment (Table 4). Three mycelial plugs (7 mm in diameter) from an actively growing colony of each fungal genotype were inoculated in each flask and incubated in the dark with constant shaking (100 rpm) at 25 °C for 7 days. An aliquot (1 mL) of *P. protegens* (strain DSMZ 13134) fresh culture was subsequently transferred into the culture broths of *Heterobasidion* spp. Co-cultures were then incubated in the dark at 4, 25, and 30 °C and harvested after 7 days. Incubation temperatures were chosen to simulate the range of temperatures at which stump treatments may be performed and hence the interaction between the bacterium and the fungal pathogens may occur. For each combination of culture medium (KB) and temperature (4, 25, and 30 °C), three conical flasks were established. Co-cultures were filtered to obtain a sample free from bacterial cells for analysis by HPLC-MS. 

### 4.4. Quantification of 2,4-DAPG, PLT, PRN Produced by P. protegens (Strain DSMZ 13134)

All reagents used for the quantification of secondary metabolites were analytical or LC-MS grade and were provided by Sigma-Aldrich (Milan, Italy). Pure 2,4-DAPG and PLT were provided by D.B.A. ITALIA (Milan, Italy), while pure PRN by Merck Life Science (Milan, Italy). The CFF resulting from cultures were purified using solid phase extraction (SPE) cartridges (Strata C18-E, 500 mg, 6 mL Phenomenex, Torrance, CA, USA). The SPE were previously activated with 3 mL of acetonitrile, washed with 2 mL of acidified water at pH 2, and the samples were then eluted to a final volume of 3 mL with a water-acetonitrile mix (1/1 volume).

The HPLC-MS system was a Varian MS-310 triple quadrupole mass spectrometer equipped with an electrospray ionization (ESI) source and 212 LC pump (Agilent, Milan, Italy). Separation was performed on a Kinetex C18 column (5 μm, 50 × 2.0 mm, Phenomenex, Torrance, CA, USA). The mobile phase solvents were water (A) and acetonitrile (B), both containing 0.1% (*v*/*v*) formic acid. The mobile phase gradient was from 90% to 10% A in 10 min (0.2 mL/min flow rate), then from 10% to 90% A in 2 min and the conditions were maintained for 3 min.

### 4.5. Inhibition of Mycelial Growth of Heterobasidion spp. by PLT

The poisoned food technique was used to assess the in vitro inhibitory activity against *Heterobasidion* spp. of secondary metabolites present in the CFF of monocultures of *P. protegens* (strain DSMZ 13134) cultured in LB broth for 24 h at 25 °C. This CFF corresponds to the CFF of *P. protegens* (strain DSMZ 13134) used in previous studies [12,13]. The only secondary metabolite detected was PLT at the concentration of 3.77 mg/L (see results); therefore, the antifungal activity of PLT was assessed on MEA amended with PLT at 3.77 mg/L concentration poured into 6 cm Petri plates. Pyoluteorin (D.B.A. ITALIA, Milan, Italy) was suspended in acetonitrile (100 mg/L) and added to the medium after autoclaving for 20 min at 121 °C, when the medium had cooled to approximately 50 °C, to yield the final concentration of 3.77 mg/L. Five genotypes for each species of *H. annosum s.l.* (Table 4) were used as target pathogens for the examination of antifungal activity of PLT. MEA plates not amended with PLT inoculated with fungal genotypes acted as controls. A MEA plug (5 mm in diameter) from an actively growing colony of each genotype was inoculated in the center of each Petri plate. Three Petri plates were prepared for each *Heterobasidion* genotype. Petri plates were incubated in the dark at 25 °C allowing a comparison with the results of the inhibition assays carried out with the raw CFF of *P. protegens* (strain DSMZ 13134) conducted under the same incubation temperature and with the same *Heterobasidion* genotypes [12].

Colony radii of *Heterobasidion* genotypes were measured (in mm) in treated (*r_T_*) plates (i.e., amended with PLT) and control (*r_C_*) plates (not amended with PLT) along two perpendicular axes after 4 and 7 days of incubation, and the two measurements for each day were averaged. As described previously [12], the MGI of *Heterobasidion* spp. was determined by calculating (in %) the radial reduction observed in treated plates in relation to the corresponding control plates with the following equation:

(2) $$\mathrm{MGI}=100\%\cdot \frac{r_{C}-r_{T}}{r_{C}}$$

### 4.6. Statistical Analyses

The average values of MGI were calculated and compared among *Heterobasidion* species for each time point (i.e., 4 and 7 days), and between time points for each *Heterobasidion* species. The above comparisons were carried out with unbiased recursive partitioning tree models [44,45] set as described in Lione et al. [46].

The same model was used to test whether the concentration of PLT (i.e., C, response variable) produced by monocultures of *P. protegens* (strain DSMZ 13134) are significantly associated with any of the following factors (i.e., covariates): culture medium, incubation temperature, and incubation period (i.e., 24 h and 7 days). The *c* statistics and its related *p*-value were calculated for each covariate [44]. Since none of the covariates was significantly associated with the response variable (see results), the concentration of PLT produced by monocultures of *P. protegens* (strain DSMZ 13134) was analyzed as such by estimating its upper tail probability distribution function. For any concentration of PLT, this function provides an estimate of the probability $\mathrm{Pr}\left(x>C\right)$ that monocultures of *P. protegens* (strain DSMZ 13134) can release PLT with a concentration x higher than a given threshold C. The equation of the function was obtained through the fit of the distribution types 0-VII included in the Pearson system of generalized frequency curves [47,48,49]. The fit was performed through maximum likelihood, and for each distribution type, the Akaike Information Criterion (AIC) was calculated [50,51]. The distribution type minimizing AIC was selected as the optimal Pearson curve [52]. The probabilities of retrieving PLT from monocultures of *P. protegens* (strain DSMZ 13134) with a concentration over 0 and up to 3.77 mg/L (i.e., mean concentration measured in the CFF, see results) were calculated from the optimal curve equation.

The concentration of PLT produced by *P. protegens* (strain DSMZ 13134) growing in co-culture with the genotype of either species of *Heterobasidion* on KB broth was analyzed through a further unbiased recursive partitioning tree model set as described above. The model was fitted to test if the production of PLT released by the bacterium in co-cultures was significantly influenced by the co-culture type (i.e., *P. protegens* (strain DSMZ 13134) and the genotype of either *H. abietinum*, *H. annosum*, *H. irregulare*, or *H. parviporum*) and the incubation temperature. 

The 95% confidence intervals (95% CI) of the average values of MGI and PLT concentration were calculated with the bootstrap bias-corrected and accelerated (BCa) method [53,54] based on the setting parameters reported in Lione et al. [46]. Statistical analyses were conducted with R version 3.6.0 [55] and with the associated packages bootstrap [56], strucchange [57], partykit [45], and PearsonDS [58]. The significance threshold was set to 0.05 for all tests.

Statistical analyses were not performed on data of concentrations of 2,4-DAPG and PRN, because such compounds were either under the LOQ or under the LOD (see results).

## 5. Conclusions

The current study revealed the presence of the antifungal compound PLT at a concentration of 3.77 mg/L in the CFF of *P. protegens* (strain DSMZ 13134). This concentration of PLT has a remarkable antifungal activity in vitro against *Heterobasidion* spp., although all lines of evidence suggest that antibiosis does not rely on a single constitutively produced metabolite, but rather on a plethora of secondary metabolites. The instability and the loss of efficacy of PLT over time may be a constraint for using this molecule as a pesticide against *Heterobasidion* spp. We did not determine the optimal fermentation conditions for the production of 2,4-DAPG, PLT, or PRN by *P. protegens* (strain DSMZ 13134), but the ability of the bacterium to produce PLT and 2,4-DAPG was demonstrated. Finally, an increased production of PLT was observed when the bacterium was grown in co-culture with *Heterobasidion* spp. at 4 °C. This finding may suggest that Proradix^®^ could perform better when stump treatments are performed at low temperatures or if the product is kept at low temperatures before use. In more general terms, the availability of further effective products for stump treatments against *Heterobasidion* species is relevant considering that the approved ones, including the chemical product urea and biological products based on *Phlebiopsis gigantea* (Fr.) Jülich, are either close to the expiration date [59] or not registered for use in several southern EU member states.

## Figures and Tables

**Table 1:** Concentration of 2,4-diacetylphloroglucinol (2,4-DAPG), pyoluteorin (PLT), and pyrrolnitrin (PRN) in monocultures of *P. protegens* (strain DSMZ 13134). Monocultures were grown in Luria–Bertani, King B, and a modified King B broth at 4 °C, 25 °C, and 30 °C and incubating them for 24 h and 7 days.

Monocultures of *P. protegens* (Strain DSMZ 13134)			Mean Concentration ± SD ^1^ (mg/L)		
Culture Medium	Incubation Temperature (°C)	Incubation Period	2,4-DAPG	PLT	PRN
Luria–Bertani broth	4	24 h	0.17 ± 0.00	0.01 ± 0.00	<LOD ^2^
		7 days	<LOD	3.63 ± 0.38	<LOD
	25	24 h	<LOD	3.77 ± 0.30	<LOD
		7 days	<LOD	0.66 ± 0.00	<LOD
	30	24 h	0.26 ± 0.00	0.81 ± 0.38	<LOD
		7 days	<LOD	0.35 ± 0.24	<LOD
King B broth	4	24 h	<LOQ ^3^	0.32 ± 0.00	<LOD
		7 days	<LOQ	8.03 ± 0.00	<LOD
	25	24 h	<LOQ	6.12 ± 0.42	<LOD
		7 days	<LOQ	2.34 ± 0.01	<LOD
	30	24 h	<LOQ	0.37 ± 0.20	<LOD
		7 days	<LOQ	0.49 ± 0.50	<LOD
Modified King B broth	4	24 h	<LOD	0.27 ± 0.00	<LOD
		7 days	<LOD	<LOD	<LOD
	25	24 h	<LOD	<LOD	<LOD
		7 days	<LOD	1.10 ± 0.02	<LOD
	30	24 h	<LOD	4.52 ± 0.49	<LOD
		7 days	<LOD	10.21 ± 3.18	<LOD

^1^ The values represent the mean and standard error of the mean for three replicates, each represents one monoculture of *P. protegens* (strain DSMZ 13134). Values were rounded to two significant digits. ^2^ <LOD means under the limit of detection, being 0.1 mg/L for every compound. ^3^ <LOQ means under the limit of quantification, being approximately 0.5 mg/L for each compound.

**Table 2:** Association between concentration of pyoluteorin (PLT) and culture conditions of *P. protegens* (strain DSMZ 13134). The *c* statistics and its *p*-value are reported for each of the factors tested for their association with the response variable. Multiple values of *c* marked by a numeric index in subscript refer to subsequent splits of the tree model. Significant *c* values (*p <* 0.05) are indicated by the symbol *.

Response Variable	Incubation Temperature	Culture Medium	Incubation Period	Co-Culture Type
Concentration of PLT produced by monocultures of *P. protegens* (strain DSMZ 13134)	*c* = 0.542	*c* = 2.132	*c* = 1.985	*-*
	*p* = 0.987	*p* = 0.718	*p* = 0.405	*-*
Concentration of PLT produced by co-cultures of *P. protegens* (strain DSMZ 13134) and *Heterobasidion* spp.	*c*_1_*=* 33.35 *	*-*	*-*	*c*_1_ = 0.626
	*p*_1_ = 1.142 × 10^−7^	*-*	*-*	*p*_1_ = 0.988
	*c*_2_ = 10.078 *	*-*	*-*	*c*_2_ = 4.754
	*p*_2_ = 2.999 × 10^−3^	*-*	*-*	*p*_2_ = 0.345

**Table 3:** Concentration of 2,4-diacetylphloroglucinol (2,4-DAPG), pyoluteorin (PLT), and pyrrolnitrin (PRN) produced in co-cultures of *P. protegens* (strain DSMZ 13134) and one genotype of different *Heterobasidion* species. Co-cultures were grown in King B broth at 4 °C, 25 °C, and 30 °C, and incubating them for 7 days.

Co-Cultures of *P. protegens* (Strain DSMZ 13134) and *Heterobasidion* spp.		Mean Concentration ± SD ^1^ (mg/L)		
Incubation Temperature (°C)	*Heterobasidion* Species	2,4-DAPG	PLT	PRN
4	*H. abietinum*	<LOQ ^2^	8.05 ± 0.01	<LOD ^3^
	*H. annosum*	<LOQ	9.14 ± 0.01	<LOD
	*H. irregulare*	<LOQ	9.90 ± 0.02	<LOD
	*H. parviporum*	<LOQ	8.28 ± 0.15	<LOD
25	*H. abietinum*	<LOQ	2.52 ± 0.02	<LOD
	*H. annosum*	<LOQ	0.84 ± 0.00	<LOD
	*H. irregulare*	<LOQ	3.31 ± 0.13	<LOD
	*H. parviporum*	<LOQ	<LOQ	<LOD
30	*H. abietinum*	<LOQ	<LOD	<LOD
	*H. annosum*	<LOQ	0.16 ± 0.18	<LOD
	*H. irregulare*	<LOQ	0.07 ± 0.12	<LOD
	*H. parviporum*	<LOQ	0.51 ± 0.30	<LOD

^1^ The values represent the mean and standard error of the mean for three replicates, each represents one co-culture of *P. protegens* (strain DSMZ 13134) and *Heterobasidion* spp. Values were rounded to two significant digits. ^2^ <LOQ means under the limit of quantification, with quantification limit being approximately 0.5 mg/L for each compound. ^3^ <LOD means under the limit of detection, with detection limit being approximately 0.1 mg/L for each compound.

**Table 4:** *Heterobasidion* genotypes used in this study. Asterisks after the accession numbers indicate genotypes selected for co-culture experiments of *P. protegens* (strain DSMZ 13134) and *Heterobasidion* spp.

MUT ^1^ Accession Number	*Heterobasidion* Species	Isolation Date	Geographic Origin
6194 *	*H. abietinum*	2016	Nus, AO, Italy
6195	*H. abietinum*	2018	Chiusa di Pesio, CN, Italy
6196	*H. abietinum*	2018	Chiusa di Pesio, CN, Italy
6197	*H. abietinum*	2018	Chiusa di Pesio, CN, Italy
6198	*H. abietinum*	2016	Chabodey, AO, Italy
3543 *	*H. annosum*	2006	Mesola, FE, Italy
1204	*H. annosum*	2005	Sabaudia, LT, Italy
3538	*H. annosum*	2006	Ansedonia, GR, Italy
3656	*H. annosum*	2006	Sabaudia, LT, Italy
6191	*H. annosum*	2015	Saint-Denis, AO, Italy
1193 *	*H. irregulare*	2005	Castelfusano, RM, Italy
1151	*H. irregulare*	2005	Sabaudia, LT, Italy
1197	*H. irregulare*	2005	Sabaudia, LT, Italy
3627	*H. irregulare*	2005	Sabaudia, LT, Italy
5666	*H. irregulare*	2006	Nettuno, RM, Italy
5612 *	*H. parviporum*	2006	Trasquera, VB, Italy
5605	*H. parviporum*	2006	Druogno, VB, Italy
5615	*H. parviporum*	1999	Charvensod, AO, Italy
6192	*H. parviporum*	2016	Chabodey, AO, Italy
6193	*H. parviporum*	2016	Chabodey, AO, Italy

^1^ MUT: Mycotheca Universitatis Taurinensis.

## Data Availability

All data are reported in the manuscript.
